# Female sex work and international sport events - no major changes in demand or supply of paid sex during the 2010 Soccer World Cup: a cross-sectional study

**DOI:** 10.1186/1471-2458-12-763

**Published:** 2012-09-11

**Authors:** Marlise Richter, Stanley Luchters, Dudu Ndlovu, Marleen Temmerman, Matthew Francis Chersich

**Affiliations:** 1 International Centre for Reproductive Health, Department of Obstetrics and Gynaecology, Ghent University, Ghent, Belgium; 2 African Centre for Migration & Society, University of the Witwatersrand, Johannesburg, South Africa; 3 School of Public Health, Faculty of Health Sciences, University of the Witwatersrand, Johannesburg, South Africa; 4 Centre for International Health, Burnet Institute, Melbourne, Australia; 5 Centre for Health Policy, School of Public Health, Faculty of Health Sciences, University of the Witwatersrand, Johannesburg, South Africa

**Keywords:** HIV, Sex work, Prostitution, Sport, South Africa

## Abstract

**Background:**

Important unanswered questions remain on the impact of international sporting events on the sex industry. Speculation about increased demand and supply of sex work often generates significant attention, but also additional funding for HIV programmes. This study assessed whether changes occurred in the demand and supply of paid sex during the 2010 Soccer World Cup in South Africa.

**Methods:**

Trained sex worker interviewers conducted face-to-face semi-structured interviews among consenting female sex workers during May-September 2010. Using bivariate analyses we compared supply, demand, sexual risk-taking, and police and health services contact pre-World Cup, to levels during the World Cup and after the event.

**Results:**

No increases were detected in indicators of sex work supply, including the proportion of sex workers newly arrived in the city (< 2.5% in each phase) or those recently entering the trade (≤ 1.5%). Similarly, demand for sex work, indicated by median number of clients (around 12 per week) and amount charged per transaction ($13) remained similar in the three study periods. Only a third of participants reported observing any change in the sex industry ascribed to the World Cup. Self-reported condom-use with clients remained high across all samples (> 92.4% in all phases). Health-care utilisation decreased non-significantly from the pre- to during World Cup period (62.4% to 57.0%; *P* = 0.075). Across all periods, about thirty percent of participants had interacted with police in the preceding month, two thirds of whom had negative interactions.

**Conclusions:**

Contrary to public opinion, no major increases were detected in the demand or supply of paid sex during the World Cup. Although the study design employed was unable to select population-based samples, these findings do not support the public concern and media speculation prior to the event, but rather signal a missed opportunity for public health action. Given the media attention on sex work, future sporting events offer strategic opportunities to implement services for sex workers and their clients, especially as health service utilisation might decrease in this period.

## Background

International sporting events are increasing in frequency and magnitude. Much media attention, especially prior to these events, highlights the presumed links between spectators and sex work during large sports tournaments [1-4]. These concerns were again raised in the recent preparations for the 2012 London Olympics [5-8]. In the months preceding the 2010 FIFA Soccer World Cup in South Africa, media speculation focused on an anticipated increase in the number of sex workers, [9] the supposed migration of sex workers to South Africa from other African countries, [1] an alleged upsurge of trafficking in women and children, [10] the increased risk of HIV transmission [9] and even that South Africa would run out of condoms [11].

During June-July 2010, about 1.4 million foreign tourists arrived in South Africa, almost a quarter travelling primarily for the World Cup [12]. South Africa has the highest number of people with HIV worldwide and carries a substantial portion of the total global burden of sexually transmitted infections (STIs) [13]. Leading health organizations, including the World Health Organization, UNAIDS and South Africa’s National Institute for Communicable Diseases urged World Cup travellers to take special precautions against STIs, including HIV [14-16].

The World Cup saw a plethora of short-term health and safer sex campaigns. There was, however, no government or FIFA-led public health programme on sex work during the World Cup, with safer sex materials distributed largely by sex work non-governmental organisations (NGOs) [17]. These included female condoms and information provided to sex workers and their clients in Cape Town. In addition, a Sex Work Crisis Helpline was established to provide information, counselling and referrals for sex workers [17].

There was little evidence to guide the health-sector response, which appears, at least in part, to have been based on speculation and public fear [18]. A number of anti-trafficking drives were initiated or expanded during this period. They were driven, amongst others, by The Salvation Army, Free Generation International, and a non-profit Christian coalition called STOP, as well as an alliance between an international network of women’s religious orders and the Southern African Catholic Bishops’ Conference [19-22].

Overall, at the time of the 2010 World Cup, little research had been conducted on the effects of major sporting events on the demand and supply of paid sex [23-25]. Existing research had focused on human trafficking for the purposes of sexual exploitation, rather than on adult, consensual sex work [26]. Evidence from the 2006 German World Cup did not support the concerns raised about human trafficking during the event [23,25]. Newspapers after the 2006 event reported that sex workers and brothel-owners were disappointed that the number of customers buying sex during that event was lower than expected [27]. While Germany legalized sex work four years before hosting the event, calls for the South African government to reform its criminal laws on sex work before the 2010 World Cup were not implemented [28].

Underpinning many of the campaigns in South Africa, was the assumption that World Cup soccer supporters (whether international tourists or locals) would require paid sex, and that this spike in demand would be matched by an increase in the supply of sex workers, or the trafficking of women and children [29]. Evidence on the impact of this event on the sex industry could assist future planning for mass entertainment events and inform better targeting of health resources and other opportunities that become available during these events. The study therefore aimed to assess whether there was a change in demand or supply of sex work during and after the South African World Cup. We also examined changes in sexual behaviour, police contact and health services for sex workers across these phases to gauge how the World Cup affected sex workers’ working conditions and interaction with services.

## Methods

Cross-sectional surveys with self-identified female sex workers were conducted in three time points. Academia-based researchers collaborated with the Sex Worker Education and Advocacy Taskforce (SWEAT) and Sisonke Sex Worker Movement (two sex worker NGOs) to identify three South African cities that hosted World Cup matches where Sisonke operated [30]. Johannesburg, the largest city in South Africa, hosted 15 matches, the most of any World Cup city [31,32]. The inner-city area of Hillbrow was selected as the research site within Johannesburg since it has a vibrant, long-standing sex trade [33-36]. Rustenburg, the second city, hosted six matches. It is in a predominantly rural province of the North West. This site comprised slums within a platinum mine area about 15 km outside Rustenburg city, the closest town to the soccer stadium. Its sex work industry mainly serves the local mining community [37]. The third site, Cape Town, was host to the second most World Cup matches (eight games). This coastal city is a popular international tourist destination [31,32] with a relatively well-documented sex work industry [38-42]. Commercial sex work was defined as ‘the exchange of sexual services for financial reward’ and only women 18 years and above were eligible [39].

SWEAT and Sisonke introduced the researchers to peer educators known to Sisonke in the three research sites who were requested to invite other peer educators or sex workers to a half-day research training workshop. Forty-five participants attended training on ethics, participant selection and interviewing. They received a certificate of attendance after workshop completion. Following role-play and review of completed study questionnaires with other workshop attendees, ten research assistants were selected in each site.

To ensure comparable procedures across phases, each research assistant was requested to administer questionnaires at the same pre-specified time of day, four days of the week, and at the identical venues as in the preceding phase. Research assistants approached every third woman known to them as a sex worker in a particular sex work venue and invited her to participate, in order to minimize bias. During each phase, research assistants administered a 43-item semi-structured questionnaire to 20 sex workers. Questionnaires were based on previous studies with sex workers in Mombasa, Kenya [43], and research on migration history and access to health care in Johannesburg [44]. Questionnaires were translated from English into four local languages (isiZulu, isiXhosa, Afrikaans and Setswana) and administered during three periods: pre-World Cup (May-early June 2010); during the World Cup (mid June- mid July 2010); and post-World Cup (September 2010). Questionnaires from the sites were couriered or personally delivered to the principal investigator (MR) for data entry at the central Johannesburg site.

### Ethical considerations

The research protocol was approved by the University of the Witwatersrand Ethics Committee (Protocol no. H100304). Sex workers willing to participate were asked to provide written informed consent. A cell-phone airtime or grocery voucher of 20 South African Rand (~US$3) was provided for time spent in the interview. Research assistants referred participants to local counselling, health and legal assistance organizations, as required. During the World Cup period, research assistants distributed female condoms and information about the toll-free sex worker helpline to participants.

All aspects of sex work are criminalized in South Africa [45] and care was taken not to collect any identifying information from participants. Data were password-protected with access restricted to the project’s data analysts.

### Study measures and statistical analysis

The impact of the World Cup was measured by comparing the characteristics of the samples we obtained in the three phases. This was based on the assumption that any changes detected in the samples would reflect the impact of the World Cup on the broader sex work population. Fluctuations in characteristics of the sex worker samples were used to indicate whether a change had occurred in the supply of sex work. Changes in sex work supply could result from an increase in the number of sex workers (more women entering sex work, movement into South Africa from elsewhere, or internal migration with women moving to the cities hosting matches) or in the time spent on sex work (increase in full-time sex workers). Indicators of sex work supply were thus: proportion of women under 24 years; nationality; arrival in the city within the last month; entry into sex work within the last month; and the proportion of sex workers who were full-time. Sex workers’ reported number of clients and the amount earned with last client specified sex work demand. Only monetary payment and not payment-in-kind was included in the analysis. To measure whether the World Cup impacted on sexual risk behaviour by sex workers, we contrasted unprotected sexual intercourse with last client, as well as whether sex workers perceived themselves to have “felt drunk” during sex with last client. Measures of heavy episodic or binge drinking (having more than five drinks every day or almost every day in the past month) are also reported. Health services contact with sex workers during the three phases was compared to assess whether there was an increase in coverage of services during the World Cup. Participants described their most recent interaction with health care in open-ended questions, which researchers classified according to type of service received. Similarly, participants were asked about contact with the police in the preceding month to assess any changes in law enforcement within the sex industry. Free text answers to police interaction in the last month were coded as a “negative interaction” if it related to police violence, arrest, harassment, theft, bribery or fines. Conversely, “positive interaction” denoted police assistance with, for example, laying a complaint or warning a participant about potential danger. Changes in supply, demand, sexual risk-taking and police and health services contact were assessed by comparing pre-World Cup indicators within these domains, to levels during the World Cup, and post-World Cup.

Participants during and after the World Cup were asked whether the event had led to changes in the sex work industry, and if so, to describe such changes in an open-ended question. Researchers coded these free-text responses as “negative” (more competition, less clients, less income, or increased police harassment); “positive” (more foreign currency, more business, could charge more, or improved relationship with police); or “mixed experiences”, both positive and negative. Finally, in the surveys during and after the World Cup, participants were asked whether they had previously completed a similar survey.

Data were entered in duplicate by separate data clerks. Following data checking and cleaning, analysis was done using Intercooled Stata, version 11 (Stata Corporation, College Station, TX, USA). Chi-square tests were used to detect differences between categorical variables. For continuous variables that had a normal distribution we used the Student’s *t* test and Anova test (for comparing means from three groups), while the Mann-Whitney U test was used for comparing data with a non-normal distribution.

## Results

Our analysis was based on 601 pre-World Cup participants, 508 during the World Cup and 538 after the event. A fifth (18.2%) of participants during the World Cup reported that they had completed a study survey previously, and a third (36.6%) noted the same post-World Cup. Table 1 shows that participants in each phased were age about 30 years on average and close to half had been in the sex work industry for five or more years. In each of the study samples, approximately two thirds were single, and 15% lived with their regular partner. Differences were detected in education level in the three samples, with fewer women sampled during the World Cup having tertiary qualifications (3.5%) compared to pre-World Cup (8.6%), though in each phase about a fifth had not completed primary school. Similarly, some differences were detected in the number of dependents and main venue where women solicited clients (higher proportion reported working in a combination of venues during and after the event). Indicators of supply and demand for sex work are presented in Table 2 and described below.

**Table 1:** Socio-demographic characteristics of female sex workers before, during and after the 2010 Soccer World Cup in three cities of South Africa

**Variables**	**Study period**			***P*-value**^**#**^
	**Pre-WC**	**During WC**	**Post-WC**	
	**(May-June; n = 601), I**	**(June-July; n = 508), II**	**(Sept; n = 538), III**	
**Age, mean yrs (sd); n**	30.3 (6.4)	30.4 (6.8)	30.2 (7.0)	0.94^¥^
	n = 599	n = 505	n = 529	
**Highest education level, n/N (%)**				
None or primary incomplete	116/572 (20.3%)	97/487 (19.9%)	105/507 (20.3%)	
Primary	298/572 (52.1%)	277/487 (56.9%)	245/507 (48.3%)	
Secondary	109/572 (19.1%)	96/487 (19.7%)	131/507 (25.8%)	
Tertiary	49/572 (8.6%)	17/487 (3.5%)	26/507 (5.1%)	0.001
**City, n/N (%)**				
Johannesburg	234/601 (38.9%)	197/508 (38.8%)	212/538 (39.4%)	
Cape Town	161/601 (26.8%)	137/508 (27.0%)	161/538 (29.9%)	
Rustenburg	206/601 (34.3%)	174/508 (34.3%)	165/538 (30.7%)	0.62
**Relationship status n (%)**				
Single	405/592 (68.4%)	336/502 (66.9%)	343/526 (65.2%)	
Regular partner, lives alone	98/592 (16.6%)	93/502 (18.5%)	107/526 (20.3%)	
Lives with regular partner	89/592 (15.0%)	73/502 (14.5%)	76/526 (14.5%)	0.62
**Number of child dependants n/N (%)**				
0	104/601 (17.3%)	125/508 (24.6%)	121/538 (22.5%)	
1	153/601 (25.5%)	145/508 (28.5%)	153/538 (28.4%)	
2-3	231/601 (28.4%)	175/508 (34.5%)	201/538 (37.4%)	
4+	113/601 (18.8%)	63/508 (12.4%)	63/538 (11.7%)	0.001
**Main venue solicits clients n/N (%)**				
Hotel/brothel or massage parlour	196/563 (34.8%)	151/472 (32.0%)	165/513 (32.2%)	
Street	198/ 563 (35.2%)	123/472 (26.1%)	126/513 (24.6%)	
Shebeen/bar/club or home	100/563 (17.8%)	40/472 (8.5%)	59/513 (11.5%)	
Combination of venues	69/563 (12.3%)	158/472 (33.5%)	163/513 (31.8%)	<0.001
**Duration in sex work industry, n/N (%)**				
<1 year	72/558 (12.9%)	81/465 (17.8%)	64/498 (12.9%)	
1–5 years	211/558 (37.8%)	165/456 (36.2%)	175/498 (35.1%)	
5+ years	275/558 (49.3%)	210/456 (46.1%)	259/498 (52.0%)	0.11

*WC* World Cup; *yrs* years; *sd* standard deviation; ^#^ chi-square test; ^¥^Anova test; Shebeen is an informal/unlicensed bar; Denominator below N in each period due to some incomplete questionnaires.

**Table 2:** Female sex work supply and demand, and changes in sexual behaviour in three South African cities during and after the 2010 Soccer World Cup using serial cross-sectional surveys

**Variables**	**Study period**			***P*-value**	***P*-value**	***P*-value**
	**Pre-WC**	**During WC**	**Post-WC**			
	**(May-June; n = 601), I**	**(June-July; n = 508), II**	**(Sept; n = 538), III**	**I vs II**^#^	**I vs III**^#^	**II vs III**^#^
**Female sex work supply**						
Proportion of women under 24, n/N (%)	107/599 (17.9%)	104/505 (20.6%)	121/529 (22.9%)	0.25	0.037	0.38
Born in South Africa, n/N (%)	358/599 (59.8%)	312/507 (61.5%)	321/536 (59.9%)	0.55	0.97	0.59
Arrival in city in last month, n/N (%)	12/548 (2.2%)	5/455 (1.1%)	2/506 (0.4%)	0.18	0.011	0.20
Entered sex work in last month, n/N (%)	7/558 (1.3%)	7/456 (1.5%)	2/498 (0.4%)	0.70	0.13	0.07
Full-time sex work, n/N (%)	431/570 (75.6%)	343/470 (73.0%)	323/505 (64.0%)	0.33	<0.001	0.02
**Female sex work demand**						
Client number, median in last week (IQR)	12 (5-20)	11 (5-20)	13 (5-23)	0.50^¥^	0.14^¥^	0.04^¥^
Amount charged with last client, median US$ (IQR; range)	13 (7-24; 0-160)	13 (7-27; 1-458)	13 (8-27; 0-200)	0.20^¥^	0.26^¥^	0.88^¥^
**Sexual risk behaviour**						
Unprotected sexual intercourse with last client, n/N (%)	29/582 (5.0%)	27/486 (5.6%)	40/524 (7.6%)	0.68	0.069	0.19
Drunk during sex with last client, n/N (%)	239/594 (40.2%)	211/483 (43.7%)	229/516 (44.4%)	0.25	0.16	0.83

WC World Cup; IQR inter-quartile range; sd standard deviation; vs versus; ^#^ chi-square test; ^¥^ Mann–Whitney U test. Denominator below N in each period due to some incomplete questionnaires.

### Sex work supply

Few differences were detected in the indicators of supply of sex work. The proportion of women under the age of 24 was 20.6% during the World Cup, comparable to before the World Cup (17.9%; *P* = 0.25), but these levels post-World Cup were 5% higher than before the event (22.9%, *P* = 0.037). Only 12.9% of participants in the pre-World Cup period had entered sex work in the last year, while 1.3% began the trade in the last month (Table 2). Similarly, only a fraction of women in the during and post-World Cup samples reported having recently begun sex work. Few had arrived in the city in the last month, though more pre-World Cup than after the event (2.4% vs. 0.4%; *P* = 0.011). No changes were detected in the proportion of non-South African women in each sample, which remained close to 40% in each phase. A similar proportion of women reporting being full-time sex workers before (75.6%) and during the World Cup (73.0%, *P* = 0.33), however, among sex workers sampled after the event, fewer reported being full-time sex workers (64%; *P* < 0.001 compared to before and *P* = 0.02 compared to after the World Cup) Similar findings were obtained when data was stratified according to city, while no significant interaction was observed within cities in supply or demand (data not shown).

### Sex work demand and sexual risk behaviour

No difference was detected in the median number of clients in the last week before and during the World Cup (12 clients and 11 clients respectively), but slightly higher client numbers were reported post-World Cup (13 clients, *P* = 0.04 comparing during and post-World Cup). The median amount charged per client did not fluctuate, remaining a median $13 per client in all phases. Also, no differences were detected in unprotected sexual intercourse with last client across the three periods, which remained below 7.6%. The proportion of women who were drunk at last sex also did not fluctuate across the samples, remaining at around 43%. Also, the proportion reporting frequent binge drinking was similar before (94/577, 16.3%) and during the World Cup (83/451, 18.4%; *P* = 0.37), but higher among women in the period after the event (118/505, 23.4%; *P* = 0.06 for the after during comparison and *P* = 0.003 comparing after and with before the event).

### Working conditions and health service utilisation

Only a third of participants (322/1046) reported that the sex industry had been altered by the World Cup (94 noting a negative change, 222 a positive change, 6 had mixed experiences). Descriptive analysis of qualitative data on these changes is presented in Table 3. In their free-text responses, a few reported observing a larger number of sex workers, while others remarked on an increase in the business and income earned. Most, however, had experienced the contrary, and noted the adverse impact of the cold weather and of supporters’ absorption with soccer matches on demand for sex work. Some said that working conditions had improved with the World Cup, such as refurbishments to the hotels where they worked and more assistance from peer educators.

**Table 3:** Female sex workers’ perceptions of changes in the sex industry due to the World Cup

**Description of changes in sex industry as a result of the World Cup (n=328)**			
Negative changes* (n=100)		Positive changes* (n=228)	
**More competition**	-“I saw a lot of new faces [female sex workers] around. There was competition. Everyone wanted to benefit” (Post-WC, CT)	**More gifts/foreign currency**	-“I did get dollars. It was R1000 [$131]. It was a lot for me. To compare the whole night of here is maybe R300 [$40]” (During WC, CT)
During WC: n=1	- Many Sowetan [urban township] girls joined the industry before the World Cup. They thought that there will be dollars, so we are now sharing our few customers. That is why we think there are no customers” (Post-WC, JB)	During WC: n=15	-“The people I sold to, one of them bought me a ticket to go to the stadium to watch soccer. It was my first time to watch soccer. I don’t even wish for that day to pass” (During WC, RB)
Post-WC: n=6		Post-WC: n=8	
**Less clients**	- “The business is not working. Our clients just come to the bar and watch the ball. There's no time for us. Even in the street there are few of them. Others there are busy watching the ball” (During WC, JB)	**More clients/business**	-“Business was burning, we made a killing” (Post-WC, CT)
During WC: n=3	- “There is no client at all because of police” (Post-WC, CT)	During WC: n=25	-“I saw a lot of change because I was able to sell a lot. So much that I wish that the World Cup could come back again so that I can buy myself two big blankets” (Post-WC, RB)
Post-WC: n=6		Post-WC: n=31	
**Less income**	-“I've seen the problem of money. We don’t have money since the World Cup started” (During WC, JB)	**Can charge more**	-“I saw change because they gave us more money than what our clients used to give us” (During WC, RB)
During WC: n=18	-“I didn’t have any money from this. Worse is to not meet one client from other country”	During WC: n=38	-“I found too much money during the World Cup. More especially when *Bafana Bafana* [South African team] was playing. I make too much money” (During WC, JB)
Post-WC: n=24	(During WC, CT)	Post-WC: n=40	
**Expectations not realised**	-“During the World Cup I was expecting too much business than ever before, but to my surprise it is only after the World Cup that I see the business improve” (Post-WC, RB)	**Meet new people**	-“I've met different people from different parts of the world. Some gave me their phone numbers and email address for networking” (During WC, JB)
During WC: n=7	-“There is no money, but they say World Cup 2010 will bring money. But I think they take money with them.” (Post-WC, JB)	During WC: n=26	
Post-WC: n=10		Post-WC: n=11	-“Mostly I met clients who treated me very differently comparing to those from here. They never treated me like a prostitute. They were very kind” (During WC, CT)
**Increased police harassment**	-“The World Cup didn’t make me happy because the police were very angry with us.” (During WC, CT	**Improved relationship: police**	-“Cops are no longer after us. They are busy with the World Cup” (During WC, JB)
During WC: n=8	-“The police harassment is too much. Every day they disturb us asking many questions and use spray guns to spray us while we are walking” (During WC, JB)	During WC: n=12	-“Because the police were not interrupting us. They were busy with drug dealers and corrupt people” (Post-WC, JB)
Post-WC: n=10		Post-WC: n=9	
**Challenging work conditions**	-“It is cold and we stand on the streets cold as it is, and leave without money. This soccer came with bad luck” (During WC, JB)	**Improved work conditions**	-“The best part was to do our business without being scared of anybody” (During WC, RB)
During WC: n=2	-“I never found many clients like before the ball started. Because people are watching the World Cup match they don’t want to come to the hotels. Some they say it is too cold” (During WC, JB)	During WC: n=2	-“The hotels have been renovated and there is more security than before” (Post-WC, JB)
Post-WC: n=0		Post-WC: n=3	-“Many peer educators from different organisations were busy distributing condoms” (Post-WC, JB)
**Personal circumstances/fears**	-“The World Cup was not so good for me because my son was very sick. I couldn’t go to work like before. It was a bad month for me” (During WC, CT	**Misc. positive remark**	-“I've seen a lot of changes during the World Cup. I'm in a hurry I can't tell you all. But the truth is I'm very excited” (During WC, JB)
During WC: n=2	-“I was scared that I might contract diseases from foreign countries which we may not have treatment for here in our country” (Post-WC, RB)	During WC: n=5	-“It was better than the normal days” (Post-WC, CT)
Post-WC: n=3		Post-WC: n=3	

*Open-ended question asked during the World Cup (During WC) and thereafter (Post-WC); CT Cape Town, multiple responses possible; JB Johannesburg; RB Rustenburg; 1USD = 7.66 South African Rand.

Table 4 shows that across each study period, about a third of participants reported contact with the police. Among those who had police contact, nearly two thirds reported this as a negative experience before the event, compared to half during or after the event (a not significant difference).

**Table 4:** Changes in police contact and provision of health services for sex workers during the World Cup study period

**Variables**	**Study period**			***P*-value**^**#**^	***P*-value**^**#**^
	**Pre-WC**	**During WC**	**Post-WC**		
	**(May-June; n = 601) I**	**(July-Aug; n = 508) II**	**(Sept; n = 538) III**	**I vs II**	**I vs III**
**Measures of police contact**					
Police contact in the last month, n/N (%)					
Any police contact	167/575 (29.0%)	144/464 (31.0%)	156/509 (30.7%)	0.49	0.56
Negative police interaction ^#^	101/575 (17.6%)	70/464 (15.1%)	80/509 (15.7%)	0.28	0.42
Positive police interaction^#^	7/575 (1.2%)	2/464 (0.4%)	5/509 (1.0%)	0.17	0.71
**Measures of health services contact**					
Health services contact in last month, n/N (%)					
Any health services contact	358/574 (62.4%)	270/474 (57.0%)	300/497 (60.4%)	0.075	0.50
Contact for physical and sexual violence*	5/574 (0.9%)	2/474 (0.4%)	6/497 (1.2%)	0.37	0.59
Contact for HIV testing*	30/574 (5.2%)	19/474 (4.0%)	15/497 (3.0%)	0.35	0.072
Contact for any STI testing including HIV*	52/574 (9.1%)	34/474 (7.2%)	26/497 (5.2%)	0.27	0.016
Contact with peer educators*	21/574 (3.7%)	5/474 (1.1%)	16/497(3.2%)	0.007	0.70

^#^ chi-square test; WC World Cup; vs versus. Denominator below 601 due to some incomplete questionnaires; ^#^ Coded from free-text descriptions that participants provided about police contact in the preceding month; *Coded from free-text descriptions that participants provided about their most recent health care experience.

Throughout the research period, just under two thirds had contact with health care in the preceding month (Table 4). The proportion of respondents who had contact with health care was 5% lower during the World Cup phase (57%) than before the event (62.4%; *P* = 0.075). Levels of contact with peer educators were disappointingly low in each period, even decreasing from 3.7% before the event to 1.1% during the World Cup (*P* = 0.007).

## Discussion

Results, based on bivariate analysis of cross-sectional surveys administered before, during and after the World Cup, do not provide evidence of large changes in sex work supply or demand in the sex workers surveyed. Few substantial changes were noted across the indicators used to assess changes in sex work supply, demand or sexual behaviour during the event. These findings echo those of a related project that entailed a three-wave cross-sectional telephonic survey of female sex workers advertising online and in local newspapers in South Africa during the same event [46]. Similarly, a before-and-after study of local sex work settings during the 2010 Winter Olympics in Vancouver found no evidence of an influx of sex workers, new sex workers, youth or reports of trafficking [47]. Unlike in our study, however, the Vancouver research team did find that sex workers perceived a heightening of police harassment during the Olympic period.

Evidence from our study does not show an increase in cross-border migrant sex workers during the World Cup, with the proportion of sex workers born in South Africa remaining about 60%. Only about one in forty sex workers reported arriving at their place of work in the last month, while even fewer had entered the industry in the past month. Similarly, the proportion of sex workers in the study samples who were below 24 years remained relatively constant throughout the research period. These findings challenge claims that that many women, particularly young women, would enter the sex industry for the World Cup. There was no change noted in the median number of clients over the World Cup period, suggesting that there was no marked spike in sex work demand. While some sex workers reported benefiting materially from the World Cup, this was not a uniform experience for all, with the median amount charged per client remaining unchanged across the research period. It is noteworthy that only a third of participants reported any change in their industry because of the World Cup. The wide range of descriptions about how the sex industry had been altered by the World Cup is striking, and suggests large variation in individual sex workers’ experiences.

Unprotected sexual intercourse with last client remained below 7.6%. It is, however, of concern that approximately 40% of sex workers reported being drunk with their last client across the phases and binge drinking was very common. This study again demonstrates the need for alcohol interventions, as a rapidly expanding body of evidence links hazardous alcohol use, especially binge drinking, with unsafe sex and HIV acquisition [48,49]. Strategies to reduce alcohol’s burden and its effects on sexual behaviour include screening for alcohol use, referrals to programmes that reduce alcohol dependence, multi-level structural interventions and sex work venue-based programmes [50,51].

Findings on police contact and high proportions of negative experiences are consistent with other studies that report frequent harassment and sex worker human rights violations by police in South Africa, [39,40,52,53] as elsewhere on the continent [54,55]. These experiences compound the marginalisation of this group, [56,57] and impact adversely on health. For example, studies in South Africa note police confiscation of condoms from sex workers as ‘evidence’ that commercial sex had taken place [38,58], while recent reports from Cape Town describe police harassment of outreach teams engaged in health promotion and HIV education with sex workers [59,60]. Police rape of sex workers has also been widely documented [38,39]. A 28 year respondent in Johannesburg in our study recounted: “'I remember when they [the police] arrested me in my hotel for loitering and they find me while I was sleeping and they rape me first before they arrest me”.

The decreased contact with health care workers during the World Cup period signals a major missed opportunity. Contact with peer educators was consistently below 4%, despite the fact that research sites were selected because of sex worker organisations or peer educator presence, and is in stark contrast with India where close to two thirds of sex workers report regular contact with peer educators [61]. This indicates the general lack of interest in sex worker support and programmes in South Africa, and specifically during the World Cup period.

In preparing for the World Cup, the then South Africa Minister of Health stated that the health sector priorities were: food safety; anti-smoking legislation; “acute cardiovascular problems resulting from stress and excitement” and disaster management [62]. In this and other documents by government officials, no mention was made of the sex industry, STIs or measures to prevent HIV transmission [63]. Calls were made to the South African government and FIFA before the World Cup to focus on sex work and to make paid sex safer, including appeals to decriminalise sex work, [64] or to implement sex work-specific health interventions in areas of concentrated sex work activity [65,66]. These were disregarded [17,67]. Only after some controversy [68], did FIFA permit the distribution of male condoms inside World Cup stadiums and Fan Parks during matches, which subsequent commentators noted were unavailable or insufficient [69]. The World Health Organization’s “Report on WHO support to the 2010 FIFA World Cup South Africa^TM^” contains no reference to “sexually transmitted infections” or “sex work”, and mentions “condoms” only once in an Appendix [70].

The study has several limitations which restrict the ability to draw inferences about the findings or to generalise them to other settings. The sampling methods used were unable to collect true population-level samples. To obtain more representative samples of this hidden population, future studies should consider employing Respondent-driven sampling (RDS) or weighting of samples [71]. Also, methods of linking participants over different time periods could be used, such as asking women to take a unique identifying number, which they then provide to an interviewer in a subsequent phase. This study was unable to link sex workers across phases and to thus account for having repeated measures on the participants who enrolled in more than one phase. We believed that collecting identifying information from participants might infringe participant confidentiality and decrease acceptability of the study, particularly at a time of reports of increased police attention to sex work [72]. Also, absence of data from clients limits the ability to draw conclusions about sex work demand. Further, use of only bivariate analyses does not enable us to control adequately for potential confounding factors. Though only self-reported data were collected, the study is strengthened by having used trained peers to conduct interview, which may have reduced the social-desirability bias in respondents’ answers. Qualitative data on World Cup changes were grouped according to themes and are represented as such, but rigorous thematic content analysis was not conducted. Although we selected three cities aiming to obtain data on diverse sex work settings, these findings may not apply to other host cities or sporting events. Research sites were purposively selected, based on the presence of sex worker advocacy groups and peer education work. Findings on the availability of health care services thus may not apply to other cities of South Africa, where sex workers may have had even lower levels of health care access. The semi-rural site may only include data on local sex work clientele as women here might not have had the same access to tourist clients as the urban sites. A further study limitation is that pre-World Cup data were collected one month before the World Cup, and thus changes in sex work supply that might have occurred prior to May 2010 in anticipation of an increase in the demand for sex work, would not have been captured. Only about 10% of sex workers had entered the trade in the year preceding the event, reassuring the research team that few long-term changes had occurred. Moreover, a longer interval between the baseline assessment and World Cup may have incurred other temporal changes (policy changes occur frequently in the country), also hindering comparison.

## Conclusion

This study found that considerable opportunities were missed to implement targeted STI and education interventions for sex workers and their clients. This oversight should be addressed in future international sporting events. Mass sport events provide health services and advocacy groups with an opportunity to capitalise on increased international scrutiny and resources to provide services for sex workers and other traditionally underserved communities.

## Competing interests

The authors declare that they have no competing interests.

## Authors’ contributions

Conceived and designed the study: MR MC SL MT. Administered the study: MR DN. Analysed the data: MR MC SL. Wrote the paper: MR MC SL DN MT. All authors read and approved the final manuscript.

## Pre-publication history

The pre-publication history for this paper can be accessed here:

http://www.biomedcentral.com/1471-2458/12/763/prepub

## References

[B1] Kwinika S Prostitutes flock to South Africa ahead of World Cup Christian Science Monitor 2010 12 May 2010. http://www.csmonitor.com/World/Africa/2010/0512/Prostitutes-flock-to-South-Africa-ahead-of-World-Cup-2010

[B2] Cooper H Ahead of World Cup, U.S. Warns Germany About Sex Trafficking New York Times 2006 6 June 2006. http://www.nytimes.com/2006/06/06/world/europe/06slavery.html?_r=1

[B3] Unknown Vancouver Winter Olympics 2010 - Prostitutes fear burnout as brothels prepare for onslaught Bild 2010 8 February 2010. http://www.bild.de/news/bild-english/news/prostitutes-fear-burnout-as-brothels-prepare-for-games-onslaught-11376172.bild.html

[B4] Associated Press Prostitution boom expected in Ukraine for Euro 2012 New York Daily News 2012 8 June 2012. http://www.nydailynews.com/sports/euro-2012/euro-2012-prostitution-boom-expected-ukraine-euro-2012-article-1.1092110

[B5] Cacciottolo M London 2012: Will the Olympics bring more prostitutes? BBC News 2012 7 June 2012. http://m.bbc.co.uk/news/magazine-18174387

[B6] Ward H The safety of migrant and local sex workers: preparing for London 2012 Sex Transm Infect 2011 87 5 368 369 10.1136/sti.2011.049825 21772039

[B7] Boff A Improving the Safety of Women: the policing of off-street sex work and sex trafficking in London 2012 Greater London Authority Conservatives, London March 2012. http://glaconservatives.co.uk/wp-content/uploads/downloads/2012/03/Report-on-the-Safety-of-Sex-Workers-Silence-on-Violence.pdf

[B8] London School of Hygiene & Tropical Medicine The Olympics and trafficking: myths and evidence 2012 LSHTM Panel Discussions, London 25 January 2012. http://www.lshtm.ac.uk/newsevents/events/2012/01/the-olympics-and-trafficking-myths-and-evidence

[B9] Khoabane P Sex trade likely to be top scorer at the World Cup Sunday Times 2009 25 October 2009. http://www.timeslive.co.za/opinion/columnists/article164721.ece

[B10] Skinner BE South Africa's New Slave Trade and the Campaign to Stop It Time 2010 18 January 2010. http://www.time.com/time/magazine/article/0,9171,1952335,00.html

[B11] Ramphekwa H Bring your own condoms, Dutch fans urged The Mercury 2009 18 November 2009. http://www.iol.co.za/index.php?art_id=vn20091118043122806C860209

[B12] South African Tourism Impact of 2010 World Cup 2010 South AfricanTourism, Sandton December 2010. http://www.southafrica.net/sat/action/media/downloadFile?media_fileid=35419

[B13] Johnson L Bradshaw D Dorrington R The burden of disease attributable to sexually transmitted infections in South Africa in 2000 South African Medical Journal 2007 97 8 Pt 2 658 662 17952222

[B14] World Health Organization, South African Department of Health Health advice for travellers to South Africa for the 2010 World Cup 2010 http://www.who.int/ith/updates/health_advice_2010_world_cup.pdf

[B15] UNAIDS Safe sex during the 2010 World Cup 2010 Geneva http://www.unaids.org/en/resources/presscentre/featurestories/2010/june/20100608worldcup/

[B16] National Institute of Communicable Diseases Travel Advisory: Communicable Diseases and 2010 FIFA World Cup, South Africa 2010 Travel, Outbreak Response, and Epidemiology and Surveillance Units of the National Institute for Communicable Diseases of South Africa, Johannesburg

[B17] Richter M Massawe D Did South Africa's soccer bonanza bring relief to sex workers in South Africa? The 2010 FIFA World Cup and the impact on sex work Agenda 2010 85 21 30

[B18] Gould C Moral panic, human trafficking and the 2010 Soccer World Cup Agenda 2010 85 31 44

[B19] Stop Human Trafficking 2010 http://2010humantraffic.org/index.htm

[B20] Free Generation International Red Card Campaign, World Cup 2010: Disqualifying Human Trafficking in Africa 2010 http://www.freegenerationintl.org/red-card-campaign

[B21] Salvation Army Salvation Army World Cup Campaign Makes a Big Noise 2010 http://www.salvationarmy.ca/2010/06/21/salvation-army-world-cup-campaign-makes-a-big-noise/

[B22] Glatz C Nuns launch new campaign against human trafficking during World Cup Catholic News Service 2010 7 May 2010. http://www.catholicnews.com/data/stories/cns/1001925.htm

[B23] The German Delegation to Multidisciplinary Group on Organised Crime / Article 36 Committee Experience Report on Human Trafficking for the Purpose of Sexual Exploitation and Forced Prostitution in Connection with the 2006 Football World Cup in Germany 2007 Council of the European Union, Brussels 5006/1/07; REV 1; CRIMORG 1; MIGR 1; 19 January 2007. http://www.legislationline.org/download/action/download/id/1499/file/16e6b168d89a7bfe7e567f7d23c9.pdf

[B24] Bowen & Shannon Frontline Consulting Human Trafficking, Sex Work Safety and the 2010. Games: Assessments and Recommendations. Report prepared for Sex Industry Worker Safety Action Group 2009 Vancouver http://vancouver.ca/police/assets/pdf/reports-policies/report-human-trafficking-2010-games.pdf

[B25] Loewenberg S Fears of World Cup sex trafficking boom unfounded The Lancet 2006 368 9530 105 106 10.1016/S0140-6736(06)68984-8 16832865

[B26] Hennig J Craggs S Larsson F Laczko F International Organization for Migration Trafficking in human beings and the 2006 World Cup in Germany IOM Migration Research Series 2007 29 http://www.humantrafficking.org/uploads/publications/mrs29-excerpt.pdf

[B27] Landler M World Cup Brings Little Pleasure to German Brothels New York Times 2006 3 July 2006. http://www.nytimes.com/2006/07/03/world/europe/03berlin.html#.

[B28] Richter ML Chersich MF Scorgie F Luchters S Temmerman M Steen R Sex work and the 2010 FIFA World Cup: time for public health imperatives to prevail Global Health 2010 6 1 1 10.1186/1744-8603-6-1 20181213 PMC2829543

[B29] SWEAT, Sisonke, UNFPA Maybe it will be better once this World Cup has passed' Research findings regarding the impact of the 2010 Soccer World Cup on sex work in South Africa 2011 Sex Worker Education & Advocacy Taskforce (SWEAT) report series, Cape Town http://sweat.org.za/index.php?option=com_k2&view=item&id=104:maybe-it-will-be-better-once-this-world-cup-has-passed-research-findings-regarding-the-impact-of-the-2010-soccer-world-cup-on-sex-work-in-south-africa&Itemid=139

[B30] Sex Worker Education & Advocacy Taskforce (SWEAT) www.sweat.org.za

[B31] Match Schedule - 2010 FIFA World Cup South Africa 2010 http://www.fifa.com/mm/document/tournament/competition/64/42/24/2010fwc_matchschedule_postevent_e.pdf

[B32] SA Cities Network State of Cities Report, 2006 2006 South African Cities Network report series, Johannesburg http://www.sacities.net/images/stories/2006/pdfs/cities_2006.pdf

[B33] Stadler J Delany S The ‘healthy brothel’: the context of clinical services for sex workers in Hillbrow, South Africa Culture, health & sexuality 2006 8 5 451 464 10.1080/13691050600872107 16923648

[B34] Wojcicki JM Malala J Condom use, power and HIV/AIDS risk: sex-workers bargain for survival in Hillbrow/Joubert Park/Berea, Johannesburg Social Science & Medicine 2001 53 1 99 121 10.1016/S0277-9536(00)00315-4 11380165

[B35] Richter M Sex work, reform initiatives and HIV/AIDS in inner-city Johannesburg African Journal of AIDS Research 2008 7 3 323 333 10.2989/AJAR.2008.7.3.9.656 25875460

[B36] Vearey J Oliveira E Madzimure T Ntini B Working the City: experiences of migrant women in inner-city Johannesburg Southern Africa Gender and Media and Diversity Journal 2011 9 228 233

[B37] Akileswaran C Lurie M Overcoming socioeconomic struggle and encountering risk: lived experiences of South African female migrants Annals of Anthropological Practice 2010 34 1 176 194

[B38] Pauw I Brener L You Are Just Whores: You Can't Be Raped': Barriers to Safer Sex Practices among Women Street Sex Workers in Cape Town Culture, health & sexuality 2003 5 465 481 10.1080/136910501185198

[B39] Gould C Fick N Selling sex in Cape Town: Sex work and human trafficking in a South African city 2008 Institute for Security Studies, Tshwane/Pretoria

[B40] Fick N Sex workers experiences with the local law enforcement in South Africa Research for Sex Work 2005 8 4 8

[B41] Fick N Enforcing Fear - Police abuse of sex workers when making arrests SA Crime Quarterly 2006 16 27 33

[B42] Fick N Sex Workers Speak Out - Policing and the sex industry SA Crime Quarterly 2006 15 March 13 18

[B43] Luchters S Chersich MF Rinyiru A Barasa MS King'ola N Mandaliya K Bosire W Wambugu S Mwarogo P Temmerman M Impact of five years of peer-mediated interventions on sexual behavior and sexually transmitted infections among female sex workers in Mombasa, Kenya BMC public health 2008 8 143 10.1186/1471-2458-8-143 18445258 PMC2397398

[B44] Vearey J Migration, access to ART, and survivalist livelihood strategies in Johannesburg African Journal of AIDS Research 2008 7 3 361 374 10.2989/AJAR.2008.7.3.13.660 25875464

[B45] Bodin C Richter M Adult, Consensual Sex Work In South Africa – The Cautionary Message Of Criminal Law And Sexual Morality South African Journal on Human Rights 2009 25 2 179 197

[B46] Delva W Richter M De Koker P Chersich M Temmerman M Sex Work during the 2010 FIFA World Cup: Results from a Three-Wave Cross-sectional Survey PLoS One 2011 6 12 e28363 10.1371/journal.pone.0028363 22163298 PMC3233553

[B47] Deering KN Chettiar J Chan K Taylor M Montaner JS Shannon K Sex work and the public health impacts of the 2010 Olympic Games Sexually transmitted infections 2012 Jun 88 4 301 303 10.1136/sextrans-2011-050235 22436199 PMC3378328

[B48] Schneider M Chersich M Neuman M Parry C Alcohol consumption and HIV/AIDS: the neglected interface Addiction 2012 Aug 107 8 1369 1371 10.1111/j.1360-0443.2012.03824.x 22703162

[B49] Chersich MF Luchters SM Malonza IM Mwarogo P King’ola N Temmerman M Heavy episodic drinking among Kenyan female sex workers is associated with unsafe sex, sexual violence and sexually transmitted infections International journal of STD & AIDS 2007 18 11 764 769 10.1258/095646207782212342 18005511

[B50] Li Q Li X Stanton B Alcohol use among female sex workers and male clients: an integrative review of global literature Alcohol Alcoholism 2010 45 2 188 199 10.1093/alcalc/agp095 PMC2842106 20089544

[B51] Kalichman SC Simbayi LC Kaufman M Cain D Jooste S Alcohol use and sexual risks for HIV/AIDS in sub-Saharan Africa: systematic review of empirical findings Prevention science: the official journal of the Society for Prevention Research 2007 8 2 141 151 10.1007/s11121-006-0061-2 17265194

[B52] Scorgie F Nakato D Ogutu AD Netshivhambe M Chakuvinga P Nkomo P Abdalla P Sibanda S Richter M “I expect to be abused and I have fear”: Sex workers’ experiences of human rights violations and barriers to accessing healthcare in four African countries 2011 African Sex Worker Alliance report series, Johannesburg http://www.plri.org/resource/%E2%80%9Ci-expect-be-abused-and-i-have-fear%E2%80%9D-sex-workers%E2%80%99-experiences-human-rights-violations-and-b

[B53] Karim QA Karim SS Soldan K Zondi M Reducing the risk of HIV infection among South African sex workers: socioeconomic and gender barriers American journal of public health 1995 85 11 1521 1525 10.2105/AJPH.85.11.1521 7485664 PMC1615708

[B54] Okal J Chersich MF Tsui S Sutherland E Temmerman M Luchters S Sexual and physical violence against female sex workers in Kenya: a qualitative enquiry AIDS care 2011 23 5 612 618 10.1080/09540121.2010.525605 21390890

[B55] Scorgie F Chersich MF Ntaganira I Gerbase A Lule F Lo YR Socio-demographic characteristics and behavioral risk factors of female sex workers in sub-saharan Africa: a systematic review AIDS and behavior 2012 16 4 920 933 10.1007/s10461-011-9985-z 21750918

[B56] Wolffers I van Beelen N Public health and the human rights of sex workers Lancet 2003 361 9373 1981 10.1016/S0140-6736(03)13594-5 12801755

[B57] Rekart ML Sex-work harm reduction Lancet 2005 366 9503 2123 2134 16360791 10.1016/S0140-6736(05)67732-X

[B58] Cassim Z Corrupt police abuse, exploit sex workers Corruption Watch 2012 29 March 2012. [ http://www.corruptionwatch.org.za/content/corrupt-police-abuse-exploit-sex-workers]

[B59] Sex Worker Education and Advocacy Taskforce HIV Plan fails sex workers SWEAT Press Release 2011 1 December. 1 December 2011. http://www.sweat.org.za/index.php?option=com_k2&view=item&id=159:nsp-ignores-evidence-and-fails-sex-workers&Itemid=136

[B60] Isaacs G Memorandum of Concern: Alleged police interference with department of health funded Outreach and Development Program for Sex Workers Letter to AIDS Directorate: Western Cape Department of Health 2011 dated 25 October 2011

[B61] Ramakrishnan L Gautam A Goswami P Kallam S Adhikary R Mainkar MK Ramesh BM Morineau G George B Paranjape RS Programme coverage, condom use and STI treatment among FSWs in a large-scale HIV prevention programme: results from cross-sectional surveys in 22 districts in southern India Sexually transmitted infections 2010 86 Suppl 1 i62 i68 10.1136/sti.2009.038760 20167734 PMC3252617

[B62] Tshabalala-Msimang M Pillay Y Preparing the health sector for the 2010 Soccer World Cup S Afr Med J 2007 97 1 40 17378280

[B63] Yancey AH 2nd Fuhri PD, Pillay Y, Greenwald I: World Cup 2010 planning: an integration of public health and medical systems Public health 2008 122 10 1020 1029 10.1016/j.puhe.2007.11.005 18313091

[B64] Richter M Chersich M Scorgie F Luchters S Temmerman M Steen R Sex work and the 2010 FIFA World Cup: time for public health imperatives to prevail Globalization and Health 2010 6 1 1 10.1186/1744-8603-6-1 20181213 PMC2829543

[B65] Richter M Massawe D A report on the consultation on HIV/AIDS, sex work and the 2010 Soccer World Cup – human rights, public health, soccer and beyond. Report on consultation convened by South African National AIDS Council & Sex Worker Education and Advocacy Taskforce Eastern Boulevard Garden Court hotel, Cape Town 26-27 November 2009. http://www.migration.org.za/report/richter-m-harper-e-masawe-d-2010-report-2010-soccer-world-cup-and-sex-work-documenting-succes

[B66] Richter M Massawe D Serious soccer, sex (work) and HIV - will South Africa be too hot to handle during the 2010 World Cup? S Afr Med J 2010 100 4 222 223 20459965 10.7196/samj.4117

[B67] Harper E Massawe D Richter M Report on the 2010 Soccer World Cup and Sex Work: Documenting Successes and Failures 2010 Sex Worker Education & Advocacy Taskforce report series, Cape Town http://www.migration.org.za/report/richter-m-harper-e-masawe-d-2010-report-2010-soccer-world-cup-and-sex-work-documenting-succes

[B68] Smith D World Cup 2010: Fifa blocking condom distribution at venues, say Aids groups Guardian 2010 4 June 2010. http://www.guardian.co.uk/world/2010/jun/04/condoms-banned-world-cup

[B69] Beres LK Missed opportunities: HIV and the 2010 FIFA World Cup Public health 2011 125 10 725 726 10.1016/j.puhe.2011.07.009 21907367

[B70] World Health Organization Report on WHO support to the 2010 FIFA World Cup South Africa™ 2011 World Health Organization, Pretoria http://www.afro.who.int/en/downloads/doc_download/6968-report-on-who-support-to-the-2010-fifa-world-cup-south-africa.html

[B71] Ramesh BM Moses S Washington R Isac S Mohapatra B Mahagaonkar SB Adhikary R Brahmam GN Paranjape RS Subramanian T Determinants of HIV prevalence among female sex workers in four south Indian states: analysis of cross-sectional surveys in twenty-three districts AIDS 2008 22 Suppl 5 S35 S44 10.1097/01.aids.0000343762.54831.5c 19098478

[B72] Mtyala Q War against sex work hots up 2009 14 December 2009. http://www.iol.co.za/news/south-africa/war-against-sex-work-hots-up-1.467664

